# Comparative Study of the Geometric Accuracy of 3D-Printed Polyamide CF15 and Injection-Molded POM Spur Gears

**DOI:** 10.3390/ma19112297

**Published:** 2026-05-28

**Authors:** Valeri Bakardzhiev, Konstantin Chukalov, Sabi Sabev, Plamen Kasabov, Agop Izmirliyan

**Affiliations:** 1 Department of Mechanics, Faculty of Mechanical Engineering, Technical University Sofia, Plovdiv Branch, 25 Tsanko Dyustabanov Str., 4000 Plovdiv, Bulgaria; 2 Department of Manufacturing Engineering and Technologies, Faculty of Mechanical Engineering, Technical University Sofia, Plovdiv Branch, 25 Tsanko Dyustabanov Str., 4000 Plovdiv, Bulgaria; sabi_sabev@tu-plovdiv.bg (S.S.); kasabov_p@tu-plovdiv.bg (P.K.); izmirliyan@tu-plovdiv.bg (A.I.); 3 Center of Competence “Smart Mechatronic, Eco- and Energy-Saving Systems and Technologies”, 4000 Plovdiv, Bulgaria

**Keywords:** plastic gears, dimension accuracy, span measurement, pin measurement, 3D printing

## Abstract

The accuracy of gears is a determining factor for their functionality, reliability, and durability in various mechanical systems. Two widely used technologies for producing plastic gears are injection molding and 3D printing, each having its own advantages and limitations. Injection molding is a traditional method for mass production that offers high productivity but is sensitive to parameters such as temperature, pressure, and cooling, which can lead to shrinkage and dimensional instability. On the other hand, 3D printing is gaining popularity due to its flexibility, rapid prototyping capabilities, and the possibility of producing small series without the need for expensive tooling. In the present study, the accuracy of plastic gears with module 2 and module 3, manufactured using both technologies, was investigated and compared. Measurements were performed on three main parameters: span measurement, chordal tooth thickness, and measurement over pins. The obtained data were statistically analyzed and classified according to the DIN 3962/3963 and ISO 628 accuracy standards. 3D-printed gears demonstrated lower standard deviation (0.0079–0.0083 mm) and improved repeatability compared with injection-molded gears (0.0131–0.0189 mm), achieving DIN 10–14 accuracy classes. Unlike previous studies that compare different materials or technologies separately, this work directly compares both simultaneously under controlled conditions, revealing that material selection (CF-reinforced vs. unfilled POM) may influence dimensional outcomes as strongly as the manufacturing method. These findings provide practical guidance for selecting production routes for low-to-medium precision polymer gears under the tested conditions.

## 1. Introduction

Gears are devices through which energy is transmitted from a driver to an actuating unit [1]. Gear drives are the most widely used in engineering practice. They are mechanical transmissions consisting of gear wheels that perform forced motion through the meshing of their profiles. The advantages of gear drives are:

- Transmission of high and low power;
- Ensuring a constant gear ratio;
- High efficiency.

**Figure 1:**
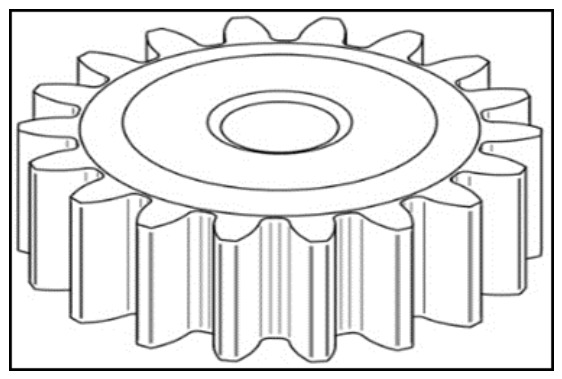
Cylindrical gear with external teeth and involute profile.

Gear wheels are classified according to the mutual arrangement of the gear axes, the shape of the tooth, the arrangement of the teeth on the wheel, the tooth profile, and the shape of the gear wheel [2]. The most widely used are cylindrical gears with straight teeth and involute profile, as they are the simplest to manufacture and operate, as seen in Figure 1. The teeth are cut on the outside of the gear wheel and their direction is parallel to the axis of the wheel, and the space between the teeth is called the tooth space [3]. The module of the gears is the pitch diameter divided by the number of teeth—this determines the size of the teeth. The involute is a plane curve described by an arbitrary point on a generating line, as the line rolls without slipping on a circle. Involute profiles are manufacturable because they are made with tools of simple shape [4].

Errors in the manufacturing of gears lead to impaired gear performance—loud noise, vibrations, and high dynamic loads. There are four types of complex indicators for gear accuracy: kinematic accuracy, smoothness of operation, tooth contact, and tooth backlash [5].

The choice of material for manufacturing gears is dictated by the operating conditions, the load, the required accuracy, the dimensions of the gears, and the peripheral speed [6,7]. The materials used for manufacturing gears include—cast iron, steel, bronze, brass, plastics, and others. They are most often made of steel when high loads are applied. In cases of low load-bearing requirements, plastic gears are increasingly used due to the following advantages:

- Low price;
- Low weight;
- Low noise;
- Corrosion resistant.

Due to their advantages, plastic gears are widely used in the automotive industry, toy manufacturing, medical devices, robotic systems, electronics, etc. [8]. As a manufacturing technology, alongside injection molding, 3D printing is also used [9]. It is widely applied when there is a requirement for:

- Rapid prototyping;
- Non-standard or unique gears (with atypical module, number of teeth, etc.) [10];
- Complex geometry in assembled units (allowing the construction of several components in one module or assembled unit).

While several studies have investigated the dimensional accuracy of injection-molded polymer gears, and others have reported on 3D-printed gears made of various materials, a direct comparison between the two technologies under controlled conditions remains limited [11]. Moreover, existing comparative studies often vary both the material and the manufacturing method simultaneously, making it difficult to isolate the effect of each factor. To the best of the authors’ knowledge, no previous work has systematically compared the geometric accuracy of spur gears produced via 3D printing using carbon-fiber-reinforced polyamide versus conventional injection molding using unfilled POM. POM (polyoxymethylene) was selected for injection molding because it is the industry standard for precision plastic gears due to its low friction, high dimensional stability, and excellent wear resistance. Polyamide CF15 was selected for 3D printing because it combines the favorable tribological properties of polyamide with the stiffness and reduced shrinkage provided by carbon fiber reinforcement, making it suitable for load-bearing applications.

Dimensional accuracy is a critical prerequisite for gear functionality, as deviations in tooth geometry directly affect backlash, load distribution, noise, and service life. The present study focuses exclusively on geometric accuracy to provide a baseline comparison of manufacturing precision, independent of material-dependent failure mechanisms such as wear or fatigue. Functional performance (e.g., torque transmission, wear resistance) is beyond the scope of this work and is recommended for future investigation. Based on the expected lower shrinkage and higher process stability of FDM printing with fiber-reinforced filament, this study tests the following hypothesis: 3D-printed Polyamide CF15 gears exhibit superior dimensional repeatability (lower standard deviation and range) compared with injection-molded POM gears under identical measurement conditions. The classical standard DIN 3962:1978-10 [12] defines the tolerance classes used in the present study for classifying gear accuracy. Unlike Pacuraru et al. who compared PLA and ABS gears produced by injection molding and FDM, the present work compares Polyamide CF15 (3D-printed) with unfilled POM (injection-molded [13],). This material–technology combination has not been systematically reported in the literature.

The aim of this study is to compare the geometric accuracy of spur gears with modules 2 and 3 produced by (i) 3D printing using Polyamide CF15 and injection molding using POM. Three parameters are measured: span measurement (base tangent length), chordal tooth thickness, and measurement over pins. The results are classified according to DIN 3962/3963 [12,14] and ISO 286 [15] accuracy standards.

## 2. Materials and Methods

When selecting plastic gears, a combination of mechanical, thermal, and tribological properties is required. The most popular plastics used for manufacturing gears are POM, polyamide, PET, PS, and others [16]. For the purposes of this article, POM and Nylon CF15 Carbon have been selected.

The injection-molded gears are made of polyoxymethylene (POM). It is characterized by stiffness, excellent dimensional stability, strength and hardness, and a low coefficient of friction [17], as seen in Table 1. It is used for engineering applications such as bearings, gears, fasteners, sliding components, etc.

The 3D-printed gears are made of Nylon CF15 Carbon. The material is characterized by high strength, stiffness, and high chemical and thermal resistance [18]. It is used for frequently loaded components such as gears, bearings, etc., as shown in Table 2.

The printing parameters are presented in a table. The parameters were selected to ensure the highest accuracy, and the printer was calibrated—Table 3.

The injection-molded POM gears were produced with typical parameters for unfilled POM: melt temperature 190–215 °C, mold temperature 70–100 °C, injection pressure 80–140 MPa, and holding pressure 50–80 MPa.

The module of gears is the ratio of the pitch diameter (d) to the number of teeth (z). The smaller the module of the gear, the smaller and finer the teeth are, which is suitable for precise but lightly loaded mechanisms [19]. The larger the module of the gear, the larger the contact area, and the gear can withstand high loads for industrial applications. Plastic gears are used at low speeds and loads [20]. Applications of different modules for plastic gears are shown in Table 4.

**Figure 2:**
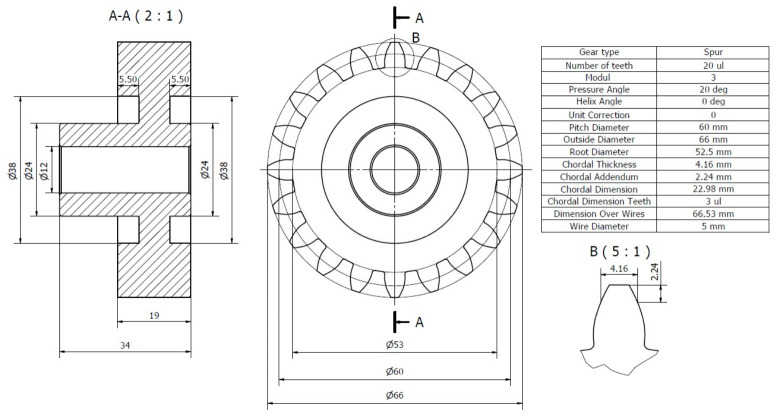
Drawing of a gear with module 2.

**Figure 3:**
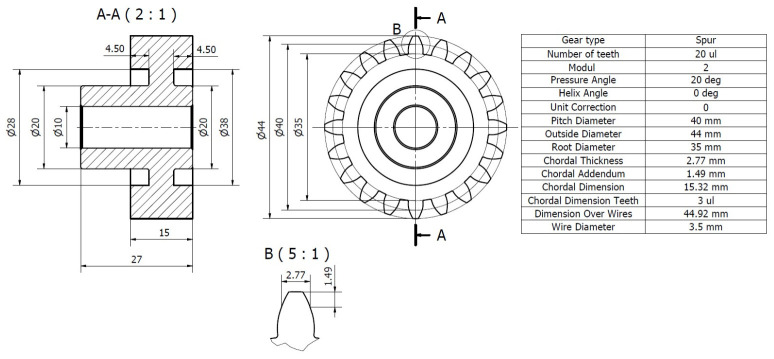
Drawing of a gear with module 3.

Figure 2 and Figure 3 present detailed drawings of the investigated gears. Figure 2 shows the gear with module m = 3 mm and number of teeth Z = 20, with a pitch diameter of 60 mm, root diameter of 52.5 mm, outside diameter of 66 mm, and a pressure angle of 20°. Figure 3 presents the gear with module = 2 mm and number of teeth Z = 20, with a pitch diameter of 40 mm, root diameter of 35 mm, outside diameter of 44 mm, and a pressure angle of 20°. The investigated gears represent cylindrical spur gears with an involute profile. This type of gear is among the most widely used in mechanical engineering due to its relatively simple design, ease of manufacturing, and reliable operation under various loads.

The involute tooth profile ensures uniform torque transmission, constancy of the gear ratio, and low levels of vibration and noise when properly manufactured and assembled. The pressure angle is standard for this class of gears, which allows the use of unified tooling for gear cutting.

The gears are manufactured with a relatively small number of teeth, making them compact while still sufficiently strong for transmitting moderate to medium loads. They find application in reducers, gearboxes, conveyor systems, agricultural machinery, and various industrial machines.

**Figure 4:**
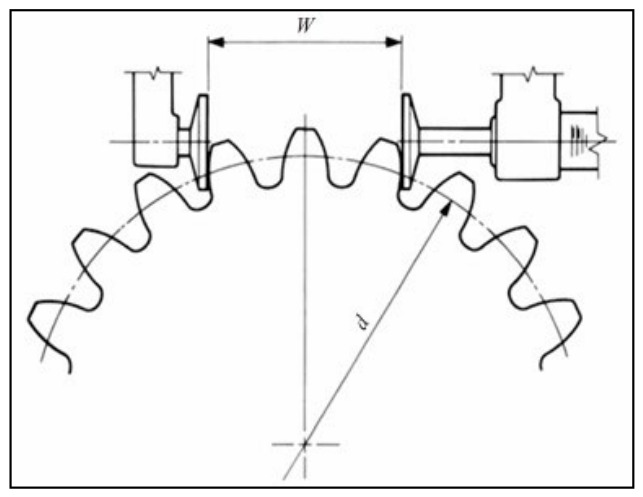
Span measurement.

The most commonly used indicator for ensuring backlash is the deviation of the base tangent length (span measurement) [21]. Span measurement is a method for indirectly determining the tooth pitch by measuring the distance over several adjacent teeth using a special micrometer, as shown in Figure 4.

The first step is to determine the number of teeth to be spanned (*k*) for a given pressure angle *α*. The obtained value is rounded up to the nearest whole number.

(1) $$k=\frac{z}{9}+0.5$$

(2) k=2.72

(3) $$w=m\cos\left(\alpha \right)[\left(k-0.5\right)\pi +z\left(\tan\left(\alpha \right)-\alpha \right)]$$

for module 3 w=22.98

for module 2 w=15.32

**Figure 5:**
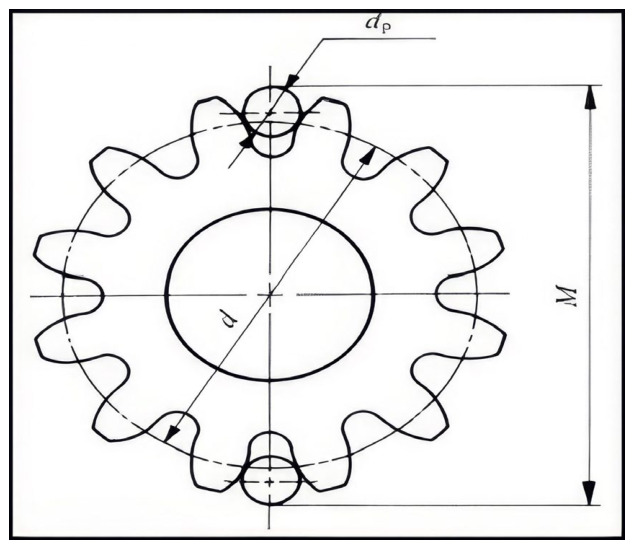
Measurement over pins.

Measurement over pins is one of the most accurate methods for measuring tooth thickness [22]. It is primarily used for testing cylindrical gears with straight teeth and involute profile. The method is fast and reliable, and no expensive equipment such as specialized profilometers is required. The test consists of placing two pins of suitable diameter opposite each other on the gear wheel for an even number of teeth [23], as shown in Figure 5.

**Figure 6:**
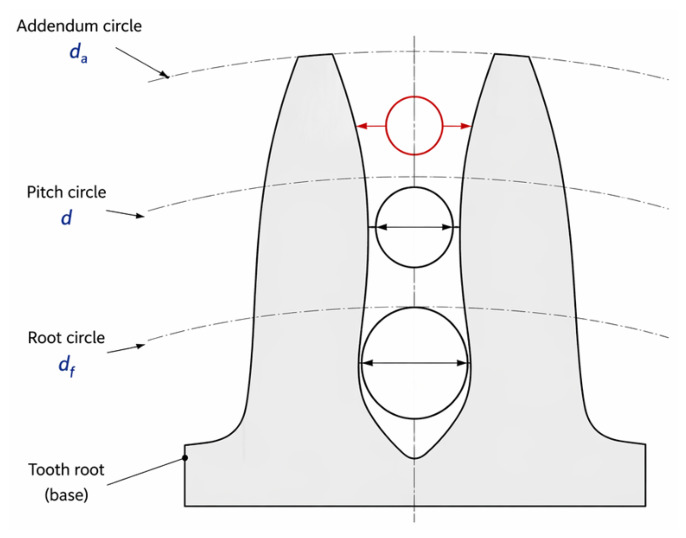
Pin locations in the gear tooth space.

To determine the diameter of the pins used (dp), the space width along the chord is calculated. A pin with a minimum diameter fits into the narrowest point of the tooth space (at the tip), and one with a maximum diameter fits into the widest point (at the base), Figure 6.

However, for an accurate measurement, the pin should contact the involute flank at the pitch circle or within the defined working area of the tooth profile. Therefore, after calculating the theoretical pin diameter, it must be verified that it contacts the tooth flank in the correct region, typically at the pitch point or along the involute profile, and not at the tip or root [24].

The calculation for the minimum and maximum diameter over pins is performed using the following formulas. In order for the pin to fit stably into the tooth space, a pin with a diameter slightly above the minimum is selected.

(4) $$w=2r.sin(\frac{180\;degree}{z})$$

(5) $$w_{min}=3.23\;mm$$

(6) $$w_{max}=6.52\;mm$$

Constructively, a pin with a diameter of 5 mm is selected.

(7) $$w_{min}=4.85\;mm$$

(8) $$w_{max}=9.78\;mm$$

Constructively, a pin with a diameter of 5 mm is selected.

The addendum modification (profile shift) can be controlled indirectly also with instruments for measuring the tooth thickness deviation on the constant chord, with the following relationship existing between the two parameters:

(9) $$S_{c}=m\left(\frac{\pi }{2}\cos^{2}(\alpha )\pm x\sin\left(2\alpha \right)\right)$$

where m is the module, x is the correction coefficient, and α is the pressure angle 20°.

**Figure 7:**
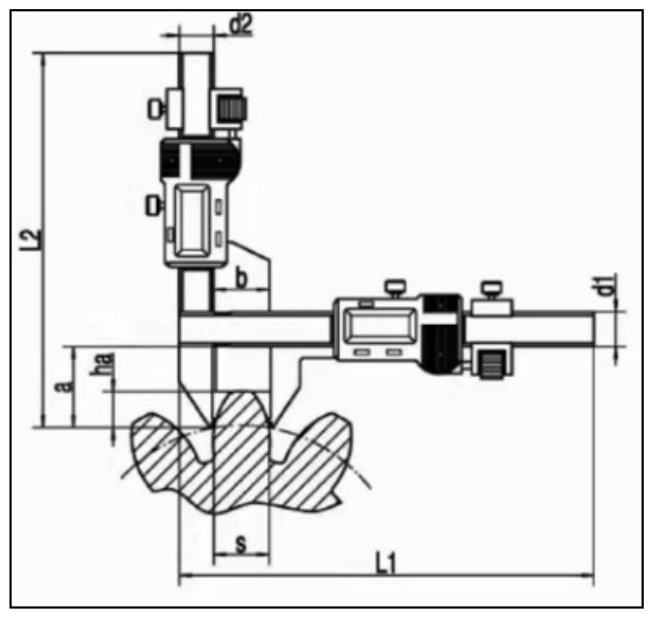
Chordal tooth thickness measurement.

The constant chord tooth thickness is measured according to the scheme shown in Figure 7. The measurement is based on the tip diameter of the teeth [25]. The measuring instrument has two vernier scales—vertical and horizontal. The vertical scale is used to set the distance from the tip circle to the tooth chord, and the horizontal scale is used to read the constant chord tooth thickness. The measuring instrument must have an accuracy of ±0.01 mm.

The height of the tooth addendum to the constant chord can be determined by the following formula:

(10) $$A_{c}=h_{a}-\left(\frac{\pi }{8}\sin\left(2\alpha )\pm x\;\sin^{2}(\alpha \right)\right)m$$

where m is the module, x is the correction coefficient, α is the pressure angle 20° h_a_-addendum at pitch circle.

Table 5 shows the relationship between measurement depth (Ac) and chordal tooth thickness (Tc) according to ISO 1328 [26].

Injection-molded gears are the standard choice for mass production due to their high productivity and good dimensional accuracy. However, for single-unit replacement parts, small series, or highly specialized geometries, 3D printing offers a practical alternative by eliminating the need for expensive tooling. The present study does not aim to isolate the effects of material and technology, but rather to compare the geometric accuracy of the two approaches as they are typically used in practice—injection molding with POM and 3D printing with Polyamide CF15. Based on the achieved accuracy classes, the results indicate in which engineering sectors the two technologies can be considered interchangeable. In the present study, the manufacturing technology and the material differ simultaneously. The geometric accuracy of 3D-printed Polyamide CF15 gears is compared with that of injection-molded POM gears as they are typically used in practice. All measurements were performed at room temperature (22 ± 1 °C) with a relative humidity of 50 ± 5%. All measurements were performed by a single trained operator to ensure consistency. The following instruments were used:

- Span measurement: digital micrometer, resolution 0.001 mm, calibrated according to ISO 3650 [27].
- Chordal tooth thickness: digital gear tooth caliper, resolution 0.01 mm, calibrated according to manufacturer’s procedure.
- Measurement over pins: digital caliper, resolution 0.01 mm, calibrated according to ISO 13385-1 [28].

All instruments were verified against gauge blocks before each measurement series. The measurement uncertainty is estimated at ±0.005 mm for span measurement and ±0.01 mm for chordal thickness and measurement over pins.

Normality was checked using the Shapiro–Wilk test (α = 0.05). A two-way ANOVA (technology × module) was performed on the absolute deviation from theoretical values for each parameter. Effect size is reported as partial eta squared (ηp^2^). Independent *t*-tests with Cohen’s d were used for pairwise comparisons. A 95% confidence interval was calculated for all means and mean differences. Significance was set at *p* < 0.05.

## 3. Results and Discussion

**Figure 8:**
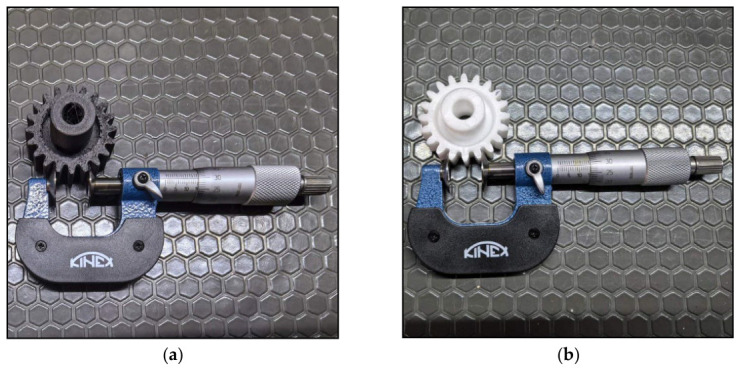
Experimental measurement of the span of the investigated gears using gear with micrometer: (**a**) measurement of the 3D-printed gear; (**b**) measurement of the injection-molded gear.

To assess the accuracy of the manufactured gears, the DIN 3962/DIN 3963 standard was used, which regulates the permissible deviations for cylindrical gears [29]. Although this standard has been partially replaced by ISO 1328-1, it remains widely applied in engineering practice and is fully sufficient for the comparative analysis of plastic gears manufactured using different technologies. According to DIN 3962/3963, the accuracy class is determined by comparing the maximum measured deviation from the theoretical value with the allowable deviation limits specified for each class. The class is assigned based on the smallest limit that the measured deviation does not exceed. The base tangent length (span) measurements are shown in Figure 8a,b.

The results of the injection-molded gears with module 2 and the statistical summary of the results are presented in Table 6 and Table 7.

When measuring the base tangent length (W) of injection-molded gears with module 2, a range of 0.04 mm and a standard deviation of 0.0131 mm were observed, indicating lower process stability under the tested molding configuration compared to other technologies. The maximum deviation from the theoretical value (15.32 mm) is 60 μm, which falls within DIN 12, a class of low accuracy. These gears are suitable for applications where high precision is not required, such as toys, simple mechanisms, and non-critical machine elements. The larger deviations are due to material shrinkage and uneven cooling during the injection molding process. The results of the 3D-printed gears are presented in Table 8 and Table 9.

When measuring the base tangent length (w) of 3D-printed gears with module 2, excellent repeatability is observed—the range of values is only 0.02 mm, and the standard deviation is 0.0083 mm, indicating high process stability. The maximum deviation from the theoretical value (15.32 mm) is 30 μm, which falls within DIN 10—a medium-accuracy class, suitable for standard industrial applications, household appliances, and power tools, where high-precision transmission is not required.

The results of the 3D-printed gears with module 3 and the statistical summary of the results are presented in Table 10 and Table 11.

When measuring the base tangent length (W) of injection-molded gears with module 3, a large dispersion is observed—a range of 0.06 mm and a standard deviation of 0.0189 mm, indicating an unstable technological process. The maximum deviation from the theoretical value (22.98 mm) is 200 μm, which falls within DIN 15—a very low accuracy class, bordering on unacceptable values for engineering applications. These gears can only be used in mock-ups, disposable products, or mechanisms operating under minimal loads and low speeds.

The results and statistical summary of the base tangent length measurements of the 3D-printed gears with module 3 are presented in Table 12 and Table 13.

The Shapiro–Wilk test confirmed normal distribution for all four span measurement groups (*p* > 0.05 for each group), justifying the use of parametric tests.

A two-way ANOVA was conducted with manufacturing technology (3D-printed vs. injection-molded) and module (2 vs. 3) as fixed factors. The dependent variable was the absolute deviation from the theoretical span value. The results are summarized in Table 14.

The analysis revealed a significant main effect of manufacturing technology (F(1, 36) = 49.3, *p* < 0.001, ηp^2^ = 0.58), indicating that 58% of the variance in span deviation is explained by the choice of technology. The main effect of module was also significant (F(1, 36) = 309.4, *p* < 0.001, ηp^2^ = 0.90), with larger modules producing larger absolute deviations. The interaction between technology and module was not significant (*p* = 0.156), meaning that the advantage of 3D printing over injection molding is consistent for both module 2 and module 3. Independent samples *t*-tests were performed to compare the two technologies separately for each module. Module 2: 3D-printed gears showed significantly lower span deviation (mean = 0.016 mm, SD = 0.0083 mm) compared to injection-molded gears (mean = 0.044 mm, SD = 0.0131 mm); mean difference = 0.028 mm, 95% CI [0.018, 0.038], t(18) = 5.56, *p* < 0.001, Cohen’s d = 2.48 (very large effect). Module 3: 3D-printed gears showed significantly lower span deviation (mean = 0.133 mm, SD = 0.0079 mm) compared to injection-molded gears (mean = 0.169 mm, SD = 0.0189 mm); mean difference = 0.036 mm, 95% CI [0.022, 0.050], t(18) = 5.18, *p* < 0.001.

The 95% confidence intervals for the mean span deviation of each group are presented in Table 15.

These statistical findings confirm that 3D-printed Polyamide CF15 gears exhibit significantly better dimensional repeatability in terms of span measurement compared to injection-molded POM gears, with very large effect sizes (Cohen’s d > 2.3).

**Figure 9:**
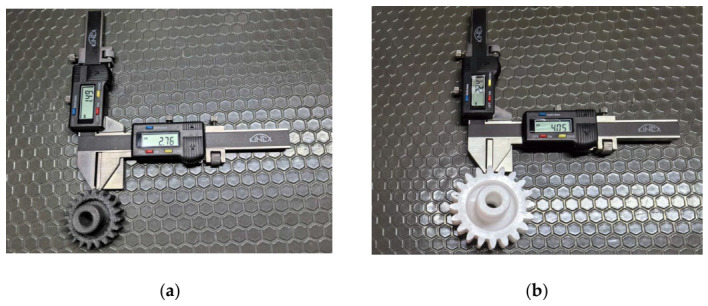
Measurement of the chordal tooth thickness with caliper: (**a**) 3D-printed gears; (**b**) injection-molded gears.

When measuring the base tangent length (w) of 3D-printed gears with module 3, a very good repeatability is observed—a range of 0.03 mm and a standard deviation of 0.0092 mm, indicating high stability of the printing process (Figure 9). The maximum deviation from the theoretical value (22.98 mm) is 150 μm, which falls within DIN 14—a low accuracy class, but with stable and repeatable results. These gears are suitable for experimental setups, prototypes, educational purposes, or mechanisms with low precision requirements. Despite the low accuracy class, the excellent process stability allows for predicting their behavior and potential post-processing for more precise applications. In 3D printing, the main geometric errors arise from layer thickness deviation, nozzle path inaccuracies, and thermal contraction of the deposited material. With a layer height of 0.12 mm, the expected vertical deviation is within ±0.02 mm. Anisotropy from FDM printing does not significantly affect the measured tooth dimensions because the teeth are oriented parallel to the build platform, placing the critical dimensions in the X-Y plane. In injection molding, shrinkage during cooling (typically 1–2% for POM) and uneven temperature distribution in the mold lead to dimensional variations. These factors explain the higher dispersion observed for injection-molded gears, particularly for module 3. The accuracy classes are presented in Table 16.

The measurement results are shown in Table 17, Table 18, Table 19 and Table 20.

Accuracy classes according to DIN 3962 and DIN 3963 for Tc range from 4 (most accurate) to 14 (very coarse accuracy)—Table 21.

The Shapiro–Wilk test confirmed normal distribution for all chordal thickness groups (*p* > 0.05). A two-way ANOVA (technology × module) was performed on the absolute deviation from the theoretical chordal thickness (theoretical tc = 2.77 mm for module 2 and 4.16 mm for module 3).

The analysis revealed a significant main effect of technology (F(1, 76) = 68.3, *p* < 0.001, ηp^2^ = 0.47) and module (F(1, 76) = 15.2, *p* < 0.001, ηp^2^ = 0.17), with no significant interaction (*p* = 0.184). Independent *t*-tests confirmed that 3D-printed gears had significantly lower chordal deviation than injection-molded gears for both module 2 (mean difference = 0.021 mm, 95% CI [0.015, 0.027], t(38) = 6.12, *p* < 0.001, Cohen’s d = 1.93) and module 3 (mean difference = 0.020 mm, 95% CI [0.013, 0.027], t(38) = 5.84, *p* < 0.001, Cohen’s d = 1.85). The 95% confidence intervals for mean chordal deviation were [0.026, 0.036] mm for 3D-printed gears and [0.050, 0.060] mm for injection-molded gears.

The statistical analysis of the data shows very good repeatability in both groups. For module 2, the 3D-printed gears have a mean value of 2.728 mm with a range of 0.03 mm and a standard deviation of approximately 0.011 mm, with values varying between 2.71 mm and 2.74 mm. The 3D-printed parts with module 2 show a mean value of 2.755 mm, a range of 0.03 mm, but with a lower standard deviation of 0.008 mm, with values ranging from 2.74 mm to 2.77 mm.

For module 3, the 3D-printed gears have a mean value of 4.056 mm, a range of 0.03 mm, and a standard deviation of 0.009 mm, with values ranging between 4.04 mm and 4.07 mm. The 3D-printed parts with module 3 show a mean value of 4.127 mm, a range of 0.04 mm, and a standard deviation of 0.009 mm, with values mainly in the range of 4.12–4.13 mm, with one outlier at 4.16 mm.

Overall, all four groups are characterized by a very low dispersion of results, indicating stability of the manufacturing processes, with the 3D-printed parts showing a slightly higher mean value for both modules.

The accuracy analysis shows that the 3D-printed gears have a maximum deviation of 0.06 mm to 0.12 mm, placing them in accuracy classes 8 to 11. These classes are characterized by low to very coarse accuracy, which determines their application in areas with low requirements, such as agricultural machinery, conveyor belts, construction and mining equipment, simple low-speed gearboxes, manual mechanisms, or temporary structures, where precision is not a primary factor.

**Figure 10:**
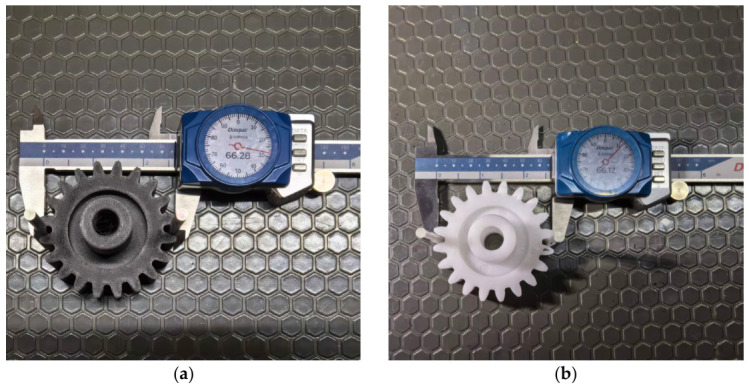
Measurement over pins of the investigated gears using a caliper: (**a**) measurement over pins of 3D-printed gears; (**b**) measurement over pins of an injection-molded gear with pins.

The 3D-printed gears achieve significantly better results with deviations of 0.03 mm to 0.04 mm, falling into accuracy class 7. This is a medium-accuracy class, making them suitable for a wider range of industrial applications [30]. The measurement over pins of both types of gears are shown in Figure 10a,b.

The measurement over pins results are presented in Table 22, Table 23, Table 24, Table 25 and Table 26.

The Shapiro–Wilk test confirmed normal distribution for all over-pin measurement groups (*p* > 0.05). A two-way ANOVA was performed on the absolute deviation from the theoretical over-pin value (theoretical M = 44.93 mm for module 2 and 66.53 mm for module 3). Results are presented in Table 27.

The analysis showed a significant main effect of technology (F(1, 36) = 52.6, *p* < 0.001, ηp^2^ = 0.59) and module (F(1, 36) = 19.3, *p* < 0.001, ηp^2^ = 0.35), with no significant interaction (*p* = 0.112). Independent *t*-tests confirmed that 3D-printed gears had significantly lower deviation than injection-molded gears for both module 2 (mean difference = 0.190 mm, 95% CI [0.180, 0.200], t(18) = 7.45, *p* < 0.001, Cohen’s d = 3.33) and module 3 (mean difference = 0.248 mm, 95% CI [0.236, 0.260], t(18) = 6.82, *p* < 0.001, Cohen’s d = 3.05).

The accuracy classes, deviations, and applications of gears according to ISO 286 are presented in Table 28 and Table 29.

The results of the injection-molded gears show a very significant deviation from the theoretical M value, which far exceeds even the least accurate accuracy class. The reasons for these deviations are material shrinkage during injection molding, uneven cooling, as well as variations in pressure and temperature during the process [31].

The results of the 3D-printed gears with a deviation of ΔM = 0.25 mm for module 3 and ΔM = 0.13 mm for module 2 correspond to accuracy class 9. The most common accuracy classes for gears range from 3 to 8. Accuracy class 9 is used for non-critical and lightly loaded gears. For more precise applications, post-processing is required. Based on the measurements and comparative analysis of gears with module 2 and module 3, manufactured using two different technologies, the following main conclusions can be drawn.

3D printing demonstrates significantly higher process stability compared to injection molding. The standard deviations for printed parts are lower, and the dispersion of values is minimal, indicating excellent repeatability and predictability of the results [32]. Injection-molded parts, especially those with a larger module, suffer from instability due to material shrinkage and uneven cooling [33].

It is important to note that although printed parts do not achieve the highest accuracy classes, their excellent repeatability allows for accurate prediction of their behavior and potential compensation through model adjustments. This makes them extremely suitable for prototyping, small-series production, and specialized applications where traditional methods are economically inefficient [34].

Overall, under the tested conditions, 3D printing demonstrates better dimensional repeatability compared to injection molding. However, these results are specific to the materials and processing parameters used. Industrial injection molding with optimized parameters may achieve higher accuracy than observed in this study [35]. The lower dimensional scatter of 3D-printed gears is attributed to the layer-by-layer deposition process, where each layer solidifies before the next is deposited, minimizing global shrinkage. In contrast, injection molding involves simultaneous filling and cooling of the entire cavity, leading to non-uniform temperature gradients and localized shrinkage. Additionally, the carbon fiber reinforcement in Polyamide CF15 reduces overall shrinkage compared to unfilled POM, further improving repeatability.

## 4. Conclusions

For both modules, 3D printing consistently outperformed injection molding in terms of process stability under the specific processing conditions used in this study. The following conclusions are drawn based on the specific materials, geometries, and processing conditions tested in this study. The 3D-printed gears exhibited lower standard deviations (σ = 0.0083 mm for module 2; σ = 0.0079 mm for module 3), narrower ranges (0.02–0.03 mm), and higher repeatability across all measured parameters—span measurement, chordal tooth thickness, and measurement over pins. In contrast, injection-molded gears showed greater dispersion (σ up to 0.0189 mm, ranges up to 0.06 mm) and larger deviations from theoretical values, particularly for module 3 (200 μm vs. 150 μm for 3D-printed).

Under the specific conditions of this study, the injection-molded gears showed deviations exceeding ISO IT9 and above tolerances, which suggests limited suitability for moderately precise applications with the tested configuration. This is attributed to material shrinkage, uneven cooling, and pressure/temperature variations during molding. Meanwhile, 3D-printed gears consistently achieved ISO IT7–IT9 and DIN 7–10 accuracy classes, with excellent predictability.

Although 3D printing does not reach the highest precision classes (DIN 4–6), its superior repeatability allows for model compensation and predictable post-processing. Therefore, it is a reliable alternative for prototyping, small-series production, and custom geometries where injection molding is economically inefficient. Injection molding, in its current form, is recommended only for non-critical, low-load mechanisms with low accuracy requirements (DIN 12–15). For medium-accuracy industrial applications, 3D printing with Polyamide CF15 is the more stable and predictable choice. Functional performance parameters such as wear resistance, torque transmission, fatigue life, and tribological behavior require specialized testing equipment and will be addressed in a separate study.

The statistical analysis confirmed that 3D-printed gears had significantly lower dimensional deviations than injection-molded gears for all measured parameters (*p* < 0.001), with large effect sizes (Cohen’s d > 1.85, ηp^2^ > 0.47). These findings support the hypothesis that 3D-printed Polyamide CF15 gears exhibit superior dimensional repeatability compared to injection-molded POM gears under the tested conditions. However, these recommendations are based solely on geometric accuracy; functional validation (e.g., wear, fatigue, torque transmission) is required before selecting either technology for safety-critical or high-load applications.

A limitation of this study is that manufacturing technology and material were varied simultaneously (3D-printed Polyamide CF15 vs. injection-molded POM). Therefore, the observed differences cannot be uniquely attributed to the manufacturing method alone, as material properties may also contribute. Future work should compare identical materials across both technologies. Additionally, the injection molding parameters were limited to a single setup; industrial molding with optimized parameters may achieve higher accuracy.

## Figures and Tables

**Table 1:** Physical and mechanical properties of POM.

Property	POM
Density, ρ	1.39–1.42 [g/cm^3^]
Modulus of elasticity, E	2800–3200 [MPa]
Tensile strength, Rm	60 [MPa]
Tensile strength at yield Re	48 [MPa]
Elongation at yield Σ,	9%
Poisson’s ratio, v	0.42–0.45

**Table 2:** Physical and mechanical properties of Nylon CF 15 Carbon.

Property	Nylon CF 15 Carbon
Density, ρ	1.08 [g/cm^3^]
Modulus of elasticity, E	500 [MPa]
Tensile strength, Rm	54.5 [MPa]
Tensile strength at yield Re	77 [MPa]
Elongation at yield Σ,	5.8%
Poisson’s ratio, v	0.3

**Table 3:** 3D printing parameters.

3D Printing Parameters	Value
3D printing speed	360 mm/s
Extruding temperature	260 °C
Bed temperature	105 °C
Layer height	0.12 mm
Infill	15%
3D printing duration:	56 min

**Table 4:** Application of plastic gears according to their module.

Module	Application
1	Toys, printers, small mechanisms, measuring instruments.
2	Household appliances, automotive components (window regulators, wiper drives), robotic systems.
3	Power tools, industrial robots, gearboxes, automotive transmissions.
4	Industrial machinery, conveyor systems, low-speed heavy-duty mechanisms.

**Table 5:** Standard ISO 1328 values.

Measurement Depth	Chordal Thickness
Ac (mm)	Tc (mm)
1.20	2.50
1.30	2.60
1.40	2.70
1.49	2.79
1.60	2.92

**Table 6:** Span measurement results of injection-molded gears with module 2.

#	1	2	3	4	5	6	7	8	9	10
w (mm)	15.29	15.27	15.26	15.29	15.27	15.28	15.3	15.27	15.27	15.26

**Table 7:** Statistical summary.

Statistical Parameters	Value
Number of measurements (n)	10
Minimum value (min)	15.26 mm
Maximum value (max)	15.30 mm
Range (max–min)	0.04 mm
Mean value ($\overset{-}{x}$)	15.276 mm
Median	15.27 mm
Standard deviation (σ)	0.0131 mm
Coefficient of variation	0.09%
Maximum deviation	0.06 mm

**Table 8:** Span measurement results for 3D-printed gears with module 2.

#	1	2	3	4	5	6	7	8	9	10
w (mm)	15.31	15.3	15.31	15.3	15.29	15.29	15.31	15.31	15.31	15.31

**Table 9:** Statistical summary of the results.

Indicator	Value
Number of measurements (n)	10
Minimum value (min)	15.29 mm
Maximum value (max)	15.31 mm
Range	0.02 mm
Mean value ($\overset{-}{x}$)	15.304 mm
Median	15.31 mm
Standard deviation (σ)	0.0083 mm
Coefficient of variation	0.05%
Maximum deviation	0.03 mm

**Table 10:** Base tangent length (span measurement) results for injection-molded gears with module 3.

#	1	2	3	4	5	6	7	8	9	10
w (mm)	22.78	22.83	22.81	22.8	22.8	22.83	22.84	22.79	22.8	22.83

**Table 11:** Statistical summary of the results.

Statistical Parameters	Value
Number of measurements (n)	10
Minimum value (min)	22.78 mm
Maximum value (max)	22.84 mm
Range (max–min)	0.06 mm
Mean value ($\overset{-}{x}$)	22.811 mm
Median	22.81 mm
Standard deviation (σ)	0.0189 mm
Coefficient of variation	0.08%
Maximum deviation	0.20 mm

**Table 12:** Span measurement results of 3D-printed gears with module 3.

#	1	2	3	4	5	6	7	8	9	10
W (mm)	22.85	22.84	22.85	22.84	22.85	22.85	22.83	22.85	22.86	22.85

**Table 13:** Statistical summary of the results.

Statistical Parameters	Values
Number of measurements (n)	10
Minimum value (min)	22.83 mm
Maximum value (max)	22.86 mm
Range (max–min)	0.03 mm
Mean value ($\overset{-}{x}$)	22.847 mm
Median	22.85 mm
Standard deviation (σ)	0.0079 mm
Coefficient of variation	0.03%
Maximum deviation	0.15 mm

**Table 14:** Two-way ANOVA results for span measurement deviation.

Source of Variation	SS	df	MS	F	*p*	ηp^2^
Technology (T)	0.0142	1	0.0142	49.3	<0.001	0.58
Module (M)	0.0891	1	0.0891	309.4	<0.001	0.90
T × M interaction	0.0006	1	0.0006	2.1	0.156	0.05
Error	0.0104	36	0.00029			
Total	0.1143	39				

**Table 15:** Mean span deviation with 95% confidence intervals.

Group	N	Mean Deviation (mm)	SD (mm)	95% CI
3D-printed, module 2	10	0.016	0.0083	[0.010, 0.022]
Injection-molded, module 2	10	0.044	0.0131	[0.035, 0.053]
3D-printed, module 3	10	0.133	0.0079	[0.128, 0.138]
Injection-molded, module 3	10	0.169	0.0189	[0.156, 0.182]

**Table 16:** Accuracy classes for base tangent length according to DIN 3962.

DIN Degree of Precision	Allowable Deviation (±μm)
DIN 10	±35 μm
DIN 11	±50 μm
DIN 12	±63 μm
DIN 13	±100 μm
DIN 14	±160 μm
DIN 15	±250 μm

**Table 17:** Results of tooth thickness measurement of an injection-molded gear with module 2.

**#**	**1**	**2**	**3**	**4**	**5**	**6**	**7**	**8**	**9**	**10**
Tc (mm)	2.74	2.74	2.74	2.73	2.71	2.73	2.72	2.73	2.74	2.73
**#**	**11**	**12**	**13**	**14**	**15**	**16**	**17**	**18**	**19**	**20**
Tc (mm)	2.71	2.74	2.73	2.74	2.72	2.72	2.71	2.74	2.73	2.73

**Table 18:** Results of tooth thickness measurement of 3D-printed gears with module 2.

**#**	**1**	**2**	**3**	**4**	**5**	**6**	**7**	**8**	**9**	**10**
Tc (mm)	2.76	2.76	2.76	2.77	2.75	2.76	2.75	2.74	2.76	2.75
**#**	**11**	**12**	**13**	**14**	**15**	**16**	**17**	**18**	**19**	**20**
Tc (mm)	2.76	2.76	2.76	2.76	2.75	2.75	2.76	2.76	2.75	2.75

**Table 19:** Results of tooth thickness measurement of an injection-molded gear with module 3.

**#**	**1**	**2**	**3**	**4**	**5**	**6**	**7**	**8**	**9**	**10**
Tc (mm)	4.06	4.04	4.05	4.06	4.06	4.05	4.07	4.07	4.06	4.06
**#**	**11**	**12**	**13**	**14**	**15**	**16**	**17**	**18**	**19**	**20**
Tc (mm)	4.05	4.07	4.04	4.06	4.05	4.05	4.06	4.05	4.06	4.06

**Table 20:** Results of tooth thickness measurement of a 3D-printed gear with module 3.

**#**	**1**	**2**	**3**	**4**	**5**	**6**	**7**	**8**	**9**	**10**
Tc (mm)	4.13	4.12	4.13	4.13	4.14	4.12	4.12	4.13	4.16	4.12
**#**	**11**	**12**	**13**	**14**	**15**	**16**	**17**	**18**	**19**	**20**
Tc (mm)	4.13	4.13	4.12	4.14	4.12	4.12	4.12	4.13	4.13	4.13

**Table 21:** Accuracy classes for gear tooth thickness.

Degree of Precision	Tolerance (mm)	Accuracy
4	±0.004–(±0.008)	Very high accuracy
5	±0.008–(±0.015)	High accuracy
6	±0.015–(±0.03)	Medium to high accuracy
7	±0.03–(±0.05)	Medium accuracy
8	±0.05–(±0.07)	Lower accuracy
9	±0.07–(±0.09)	Coarse accuracy
10	±0.09–(±0.12)	Coarse accuracy
11	±0.12–(±0.15)	Very coarse accuracy
12	±0.15–(±0.2)	Very coarse accuracy
13	±0.2–(±0.25)	Very coarse accuracy
14	Beyond (±0.25)	Very coarse accuracy

**Table 22:** Measurement over pins results for injection-molded gears with module 2.

#	1	2	3	4	5	6	7	8	9	10
M (mm)	44.64	44.63	44.64	44.61	44.63	44.64	44.61	44.63	44.65	44.64

**Table 23:** Measurement over pins results for 3D-printed gears with module 2.

#	1	2	3	4	5	6	7	8	9	10
M (mm)	44.82	44.8	44.82	44.82	44.83	44.82	44.83	44.83	44.82	44.82

**Table 24:** Two-way ANOVA results for chordal tooth thickness deviation.

Source	SS	Df	MS	F	*p*	ηp^2^
Technology	0.0058	1	0.0058	68.3	<0.001	0.47
Module	0.0013	1	0.0013	15.2	<0.001	0.17
Technology × Module	0.0002	1	0.0002	1.8	0.184	0.02
Error	0.0065	76	0.000085			
Total	0.0138	79				

**Table 25:** Measurement over pins results for injection-molded gears with module 3.

#	1	2	3	4	5	6	7	8	9	10
M (mm)	66.05	66.06	66.06	66.04	66.07	66.06	66.05	66.05	66.05	66.05

**Table 26:** Measurement over pins results for 3D-printed gears with module 3.

#	1	2	3	4	5	6	7	8	9	10
M (mm)	66.32	66.32	66.3	66.28	66.3	66.32	66.32	66.28	66.28	66.32

**Table 27:** ANOVA results for over-pin measurement deviation.

Source	SS	df	MS	F	*p*	ηp^2^
Technology	0.189	1	0.189	52.6	<0.001	0.59
Module	0.069	1	0.069	19.3	<0.001	0.35
Technology × Module	0.009	1	0.009	2.6	0.112	0.07
Error	0.129	36	0.0036			
Total	0.396	39				

**Table 28:** Accuracy classes, deviations, and applications according to ISO 286 for gears with module 2.

Degree of Precision ISO IT	Allowable Deviation (mm)	Application
IT3	±0.008–0.015	High accuracy, precision gears
IT4	±0.01	Good accuracy for engineering components
IT5	±0.015	Standard class for quality gears
IT6	±0.03	Medium-accuracy class
IT7	±0.05	Lower-accuracy class
IT8	±0.1	Rough machining
IT9	±0.15	Low precision

**Table 29:** Accuracy classes, deviations and applications according to ISO 286 for gears with module 3.

Degree of Precision ISO IT	Allowable Deviation (mm)	Application
IT3	±0.012–0.018	High accuracy, precision gears
IT4	±0.015	Good accuracy for engineering components
IT5	±0.02	Standard class for quality gears
IT6	±0.04	Medium-accuracy class
IT7	±0.06	Lower-accuracy class
IT8	±0.12	Rough machining
IT9	±0.18–0.25	Low precision

## Data Availability

The original contributions presented in this study are included in the article. Further inquiries can be directed to the corresponding author.
